# Evidence and consequences of self-fertilisation in the predominantly outbreeding forage legume *Onobrychis viciifolia*

**DOI:** 10.1186/s12863-015-0275-z

**Published:** 2015-10-07

**Authors:** Katharina Kempf, Christoph Grieder, Achim Walter, Franco Widmer, Sonja Reinhard, Roland Kölliker

**Affiliations:** Molecular Ecology, Agroscope Reckenholz ISS, Reckenholzstrasse 191, 8046 Zurich, Switzerland; Fodder Plant Breeding, Agroscope Reckenholz ISS, Reckenholzstrasse 191, 8046 Zurich, Switzerland; Crop Science, ETH Zurich, Universitätstrasse 2, 8092 Zurich, Switzerland

**Keywords:** *Onobrychis viciifolia*, Sainfoin, Self-fertilisation, Inbreeding depression, SRAP marker, SSR marker, Tetraploidy, Outbreeding

## Abstract

**Background:**

Sainfoin (*Onobrychis viciifolia*) is a promising alternative forage plant of good quality, moderate nutrient demand and a high content of polyphenolic compounds. Its poor adoption is caused by the limited availability of well performing varieties. Sainfoin is characterised as tetraploid and mainly outcrossing, but the extent of self-fertilisation and its consequences was not investigated so far. This study aimed at assessing the rate of self-fertilisation in sainfoin under different pollination regimes and at analysing the consequences on plant performance in order to assist future breeding efforts.

**Methods:**

The self-fertilisation rate was assessed in three sainfoin populations with artificially directed pollination (ADP) and in three populations with non-directed pollination (NDP). Dominant SRAP (sequence-related amplified polymorphism) and codominant SSR (simple sequence repeats) markers were used to detect self-fertilisation in sainfoin for the first time based on molecular marker data.

**Results:**

High rates of self-fertilisation of up to 64.8 % were observed for ADP populations in contrast to only up to 3.9 % for NDP populations. Self-fertilisation in ADP populations led to a reduction in plant height, plant vigour and, most severely, for seed yield.

**Conclusions:**

Although sainfoin is predominantly outcrossing, self-fertilisation can occur to a high degree under conditions of limited pollen availability. These results will influence future breeding efforts because precautions have to be taken when crossing breeding material. The resulting inbreeding depression can lead to reduced performance in self-fertilised offspring. Nevertheless the possibility of self-fertilisation also offers new ways for hybrid breeding based on the development of homogenous inbred lines.

**Electronic supplementary material:**

The online version of this article (doi:10.1186/s12863-015-0275-z) contains supplementary material, which is available to authorized users.

## Background

Legumes are particularly valuable components of permanent and temporary grasslands, as they increase forage yield and quality and simultaneously decrease the need for nitrogen fertilisation through symbiotic N_2_ fixation [1]. The perennial legume sainfoin (*Onobrychis viciifolia*) combines a multitude of positive characteristics of grassland legumes. It is adapted to drought prone areas and few important pests and pathogens are reported for this species [2]. The name sainfoin is derived from the French words “sain” and “foin” which means “healthy hay” and implies the health-promoting features of this species. Sainfoin is characterised by high contents of condensed tannins which, at a moderate level, support protein digestion and help to reduce bloat in sheep [3, 4] or cattle [5]. Tannins are also valued for their anti-parasitological effects against gut parasites [6]. Feeding sainfoin may help to reduce the use of medications in animal husbandry.

The use of sainfoin in ruminant nutrition is focused on roughage production in pure or mixed stands. For this, predominantly tetraploid varieties (2n = 4x = 28) are used, but diploid populations (2n = 2x = 14) also exist in natural grasslands. Based on comparative cytological studies, an autopolyploid inheritance was suggested for sainfoin [7]. This was verified by a preponderance of tetrasomic gene segregation, which is characteristic for autotetraploid species, as shown in a study based on isozyme variation [8]. However, the latter study also found some evidence for disomic segregation and some authors have suggested an allopolyploid condition of sainfoin, although no direct evidence was given [9]. Sainfoin is insect-pollinated with six insect species acting as pollinators [10], i.e. bumble bees (*Bombus huntii* Greene¸ *B. occidentalis* Greene, *B. rufocinctus* Cress and *B. fervidus*), honey bees (*Apis mellifera* L.) and alfalfa leafcutter bees (*Megachile rotundata*). Sainfoin was described to be mainly cross-fertilising [11, 12], but a gametophytic or sporophytic self-incompatibility has not been described. Cross-pollination may be mediated by the architecture of the flower, where the position of pistil and anthers could prevent self-pollination [13–15]. However, self-fertilisation has been observed to a certain extent [15–18]. Demdoum [15] verified that pollen tube growth occurred after self-pollination, but directed self-pollination by hand resulted in only small numbers of seeds.

Sainfoin was traditionally widely used in monoculture for hay production and is nowadays mostly used as a component of mixed meadows in extensive agriculture. Although forage yield and quality as well as animal health supporting properties make sainfoin an ideal choice for ruminant forage production, sainfoin is not widely adopted in today’s agriculture. The poor adoption is mainly due to lower forage yield compared to other legumes [19], a low persistency mostly due to a poor adaptation to wet areas and cold winters [20], and a weak competitive ability against other species [21]. These disadvantages lowered the interest in sainfoin and breeding efforts have been reduced in the last 30 years to a minimum. Consequently, there is a general lack of well adapted varieties. In the plant variety catalogues & databases of the EU [22] only 22 sainfoin varieties are listed, compared to 218 and 385 varieties for red clover (*Trifolium pratense L.*) and alfalfa (*Medicago sativa* L.), respectively. In addition, seed of sainfoin varieties is often scarce due to low seed yield, which impairs seed multiplication. Another reason for the low breeding progress in sainfoin might be the still limited knowledge on the genetics of this species [23]. The majority of sainfoin varieties are developed as population or synthetic varieties and hence comprise a wide range of different heterozygous genotypes. The amount of gene heterogeneity in such populations allows on the one hand adaptation to diverse environmental conditions. On the other hand, deleterious alleles are hidden in such populations and could emerge after some generations. In other species such as maize (*Zea mays* L.), sugar beet (*Beta vulgaris* L.) and rye (*Secale cereale* L.), breeding success was accelerated by the development of hybrid varieties, which exploit heterosis [24–26]. Hybrid varieties are based on a pair-cross between two homozygous plants with different genetic background. The combination of favourable alleles in the offspring leads to increased performance known as hybrid vigour. Hybrid breeding is so far not considered for sainfoin due to the outbreeding fertilisation system and the resulting difficulties of producing homozygous parental plants. Assessing the rate of self-fertilisation and its consequences in sainfoin will indicate, whether the development of inbred lines for hybrid breeding is feasible.

The rate of self-fertilisation may be directly influenced by pollen availability through crossing partners, mainly depending on the amount of simultaneously flowering individuals of the same species [27]. Pollen availability may be markedly different in natural conditions in the field than under controlled conditions such as in breeding nurseries or pollination cages. However, detailed information on self-pollination rates under different conditions is not available for sainfoin, partially due to the lack of large scale availability of sequence specific molecular markers. Marker systems not relying on *a priori* sequence information are applicable to a wide range of species and therefore may offer a means to study self-fertilisation in sainfoin. The sequence-related amplified polymorphism (SRAP) marker technique relies on the amplification of GC rich regions of the genome and produces dominant markers that can be distinguished based on different amplicon lengths [28].

A major limitation associated with self-pollination in predominantly outbreeding species is the decrease in plant performance and fitness associated with inbreeding depression, i.e. the accumulation of deleterious alleles in the progeny. Knowledge on the extent of inbreeding depression following self-fertilisation in a species is important for breeding decisions such as the selection of parental plants for bi- or multiparental crosses or the development of homozygous lines for hybrid breeding.

The main objective of this study was to increase the knowledge on the extent of self-fertilisation in sainfoin and its consequences on plant performance and fitness in order to provide the basis to optimise breeding strategies for the development of better varieties and to promote a wider adoption of sainfoin cultivation. In particular, we aimed at developing a method to assess self-fertilisation in sainfoin with dominant SRAP (sequence-related amplified polymorphism) and co-dominant SSR (simple sequence repeats) markers. This method was used to compare the extent of self-fertilisation under two pollination regimes, i.e., artificial directed and natural non-directed pollination. Based on the identification of self-fertilised offspring and non-self-fertilised offspring, the effect of inbreeding on agronomic traits such as plant height, plant vigour, flowering time and seed development was analysed.

## Methods

### Plant material and field trial

Three populations of sainfoin (*Onobrchis viciifolia*) generated through artificially directed pollination in the greenhouse (ADP) and three populations generated under non-directed pollination (NDP) in the field were examined for rates of self-fertilisation. For generation of ADP populations, plants from the four varieties *O. viciifolia* ‘Visnovsky’, ‘Brunner’, ‘Perly’ and ‘Perdix’, which differ for origin, flowering time, growth habit and mean vigour, were selected [29, 30]. All varieties were of the multiple flowering type *bifera*, which shows a fast development with flower emergence in the year of sowing and restart of flowering after cutting [31]. Five clones were established from each of six sainfoin plants via stem cuttings, which were placed in wet soil without adding growth promoting substances. Cuttings were covered with plastic foil for two weeks to preserve humidity and established plants were grouped pairwise in separate greenhouse chambers for seed production (Table 1).

**Table 1:** Overview of populations derived from artificially directed pollination (ADP) and non-directed pollination (NDP)

ADP populations	Plants (total)	Parent 1	Parent 2	NDP populations	Plants (total)	Maternal parent
ADP 1	145	Visnovsky_1^a^	Perly_1^c^	NDP 1	103	Perly^c^
ADP 2	218	Visnovsky_2^a^	Perly_2^c^	NDP 2	109	Perdix^c^
ADP 3	237	Brunner_1^b^	Perdix_1^c^	NDP 3	110	Perly^c^

^a^Agrogen, spol. s.r.o., Troubsko, Czech Republic

^b^Agroscope, Zurich, Switzerland

^c^Agroscope, Nyon, Switzerland

Artificially directed pollination were conducted in January 2012 by placing bumble bee (*Bombus terrestris L.*) hives (“*Bombus Maxi Hummeln”,* Andermatt Biocontrol, Switzerland) into each chamber for three weeks. Seeds from successful pollinations were germinated in May 2012 on moistened filter paper in petri dishes at 20 °C under normal daylight [32]. The final number of offspring per ADP population varied from 145 to 237 (Table 1). The seedlings were transferred to turf pots and nursed in the greenhouse for two months. In July 2012, the plants were planted at the field site in Delley (Delley, Fribourg, Switzerland) with a distance of 50 cm between plants. Plants were arranged in two rectangular blocks, both with an equal proportion of plants originating from each cross to balance potential environmental effects. Within blocks, plants were randomly arranged in rows each consisting of ten offspring of the same maternal plant.

Populations based on naturally non-directed pollination (NDP) were selected from three different field sites of rectangular shape. The site of NDP 1 was a mixed meadow containing the sainfoin variety Perly located in an urban area in Zurich (Zurich, Switzerland). Sites of NDP 2 and NDP 3 were seed multiplication trials for the *O. viciifolia* varieties Perdix and Perly, both located in a rural area in Delley (Delley, Fribourg, Switzerland). Maternal plants were identified at eight positions in each field, which were chosen at the corners and in the middle of the field for NDP 2 and NDP 3. Plant material was sampled from these plants for DNA extraction and seeds were harvested and germinated in the greenhouse to build up the three NDP populations (Table 1).

Sites for the field trial and for sampling plant material were provided by DSP Delley seeds and plants Ltd (Delley, Fribourg, Switzerland; ADP1-3, NDP2-3) and Agroscope, ISS (Zurich, Switzerland; NDP1).

### Phenotyping of ADP populations

Traits associated with agronomic performance were assessed in the first main season in 2013 on a single plant basis. Plant height was measured in summer 2013 (length of stretched plants from base to the last leaflet). The Plant vigour, was visually scored on a scale from 1 (weak) to 9 (strong) in summer 2013. Flowering time was determined in days after first of May 2013 when a plant showed at least three open flowers [30]. In the first main season, seed number and weight were assessed by destructive harvest. As sainfoin seeds ripen time-delayed from the base to the top of the inflorescence, the risk to loose seeds before full maturity of all seeds is high [33]. To reduce possible loss of seeds, tillers carrying seeds were cut 10 cm above ground in July 2013 and directly put into cotton bags. After drying at 30 °C for two days, plants were threshed manually to avoid seed damage that might interfere with seed counting. Seeds were separated from the plant material by rough sieving (5 mm grid size), followed by cleaning with an air separator (Kurt Pelz Maschinenbau, Bonn, Germany) and fine sieving (1.6 mm grid size). Cleaned seeds were then counted and weighed on a single plant basis.

### DNA extraction and marker genotyping

Fresh leaf material from ADP was sampled in October 2012 and from NDP in July 2013. Afterwards, the plant material was freeze dried over a period of 48 h. The dried plant material was then ground with a ball mill (Cell tissue Analyzer 2, Quiagen, Hilden, Germany) for subsequent DNA extraction using the illustra^TM^ DNA Extraction Kit PHYTOPURE (GE Healthcare, Little Chalfont Buckinghamshire, United Kingdom) following the manufacturer’s instructions. The DNA concentration was determined by gel electrophoresis with a mass standard (High DNA Mass Ladder, Invitrogen™, Life Technologies, Carlsbad, USA). Marker genotyping was performed using dominant sequence-related amplified polymorphism (SRAP) markers [28] and two co-dominant SSR markers (“personal communication”, M. Mora Ortiz, National Institute of Agricultural Botany, NIAB, UK). Four fluorescently labelled forward and reverse primers [me1 to me4 and em1 to em4; Table 2; [28]] were used in 16 combinations in the parental plants and offspring of the ADP populations and in eight combinations in the maternal plant and offspring of the NDP populations (Additional file 1: Table S1). The PCR reactions were performed using an iCyler (Biorad, Hercules, USA) with a sample volume of 20 μL, each containing 10 ng DNA template, 1 × Go Taqflexi buffer (Promega, Madison, USA), 3 mM MgCl_2_ (Promega), 0.2 mM dNTPs (Promega), 0.2 μM fluorescently labelled forward primer, 0.2 μM reverse primer and 0.5 U polymerase G2 (Promega). The PCR conditions consisted of 5 min at 94 °C, followed by 5 cycles of 94 °C for 1 min, 35 °C for 1 min and 72 °C for 1 min, followed by 35 cycles at 94 °C for 1 min, 50 °C for 1 min and 72 °C for 1 min. The reaction ended with 7 min at 72 °C [28]. For fragment analysis, 1 μL of the undiluted PCR product was mixed with 0.5 μL LIZ 600 (GeneScan^TM^-600LIZ® Size Standard; AB applied biosystems, Forster City, USA) and 10 μL Formamide (Hi-Di™ Formamide; AB, applied biosystems) in a 384 well plate and heated for 5 min at 94 °C. After cooling down, samples were analysed with an Applied Biosystems 3500/3500XL Genetic Analyzer. Resulting SRAP fragments were scored for presence or absence of marker alleles using GeneMarker (Softgenetics, V2.4.0 Inc., State College, USA). To allow for the distinction between cross- and self-fertilisation in ADP populations, only marker alleles present in one parent and absent in the other parent (nulliplex alleles) were recorded. For NDP populations, only fragments which were absent in the maternal plant and present in at least one of the offspring were scored. In addition to the SRAP markers, two previously developed unpublished co-dominant SSR markers were used with the same DNA samples (Table 2). PCR reactions were conducted in a volume of 20 μL, containing 10 ng DNA, 1 × Go Taqflexi buffer (Promega, Madison, USA), 2.5 mM MgCl_2_ (Promega), 0.2 mM dNTPs (Promega), 0.2 μM fluorescently labelled forward primer, 0.2 μM reverse primer and 0.5 U Polymerase G2 (Promega), using conditions as for a touchdown PCR with 4 min at 94 °C, 12 cycles of 30 s at 66 °C with - 1 °C decrease at each cycle plus 30 s at 72 ° C, and 30 cycles of 30 s at 94 °C, 30 s at 54 °C plus 30 s at 72 °C, followed by 7 min at 72 °C. Fragment analysis was performed as described for SRAP markers.

**Table 2:** SRAP and SSR primers used to determine the rate of self-fertilisation

Marker	Forward primer (5′–3′)		Reverse primer (3′–5′)		Reference
SRAP	me1	TGAGTCCAAACCGGATA	em1	GACTGCGTACGAATTAAT	Li and Quiros, 2001 [28]
SRAP	me2	TGAGTCCAAACCGGAGC	em2	GACTGCGTACGAATTTGC	Li and Quiros, 2001 [28]
SRAP	me3	TGAGTCCAAACCGGAAT	em3	GACTGCGTACGAATTGAC	Li and Quiros, 2001 [28]
SRAP	me4	TGAGTCCAAACCGGACC	em4	GACTGCGTACGAATTTGA	Li and Quiros, 2001 [28]
SSR	OVLegPl17_F	GGGTGTTAGTTATCCATTTCC	OVLegPl17_R	ACATACTAGCCTTCTGGGGTA	Mora Ortiz, “pers. comm”
SSR	OVLegPl27_F	AATGGAATCTCGGAGACAG	OVLegPl27_R	GGAAGAAGACGAAGTAGTAGGA	Mora Ortiz, “pers. comm”

### Detection of self-fertilisation

In ADP populations, an offspring was considered the result of a self-fertilisation (selfing) when all marker alleles scored as absent in one parent (nulliplex) were also absent in the offspring. All remaining offspring were classified as the product of a cross-fertilisation (crossings). The SRAP marker data were additionally used for a principle component analysis (PCA) to visualise a clustering dependent on origin of cross- or self-fertilisation. For comparison, PCA was also performed on simulated data representing 200 dominant marker scores for two heterozygous parents and 50 self-fertilised and 50 cross-fertilised progeny per parental plant (Additional file 2: Sheet S1). For SSR markers, all offspring containing marker alleles that were unique to the pollen donor plant (i.e. not present in the maternal plant) were classified as crossings. SSR data was used to complement the results from the SRAP analysis. In NDP populations, offspring with SRAP and SSR marker alleles not present in the maternal plant were classified as crossings, whereas the remaining offspring were considered as putative selfings.

### Statistical analysis

Phenotypic data of ADP populations were analysed on a single plant basis using general linear models to assess the effect of population, parental plant and breeding type of offspring (selfing vs. crossing) on plant height, seed yield, plant vigour and flowering time:

$$ {y}_{ikj}=\mu +{p}_i+p{g}_{ik}+p{b}_{ij}+ pg{b}_{ikj}+{e}_{ikj}, $$

where μ is the general intercept, *p*_*i*_ is the effect of the i^th^ ADP population, *pg*_*ij*_ the effect of the k^th^ parent and *pb*_*ij*_ the effect of the j^th^ breeding type, both nested within the i^th^ ADP population, *pgb*_*ijk*_ the effect of the j^th^ breeding type nested within the k^th^ parent and i^th^ population, and *e*_*ikj*_ is the residual error.

Effects of NDP population and sampling position on the self-fertilisation rate were analysed with generalized linear models using the following logistic regression model

$$ \mathrm{logit}\left[\mathrm{P}\left(\mathrm{Self}-\mathrm{Fert}\right)\right] = \mu +{p}_i+{s}_{ij}, $$

where logit[P(Self-Fert)] is the logit of the self-fertilisation rate, *μ* is the general intercept, *p*_*i*_ is the effect of the i^th^ population and *s*_*ij*_ is the effect of the j^th^ sampling position within the i^th^ population. Because sampling positions “corner” and “middle” were only applied for NDP 2 and NDP 3, NDP 1 was excluded from this analysis and a further model, reduced by the sampling position term (*p*_*i*_), was applied on total numbers per population only to test for differences among the three populations.

All statistical analyses and calculations were performed within the R-environment (R Core Team, 2014), using functions prcomp() for principal components analysis of SRAP marker data, lm() for general linear models for analysis of phenotypic data and glm() for generalized linear models.

## Results

### Self-fertilisation in ADP populations

**Fig. 1:**
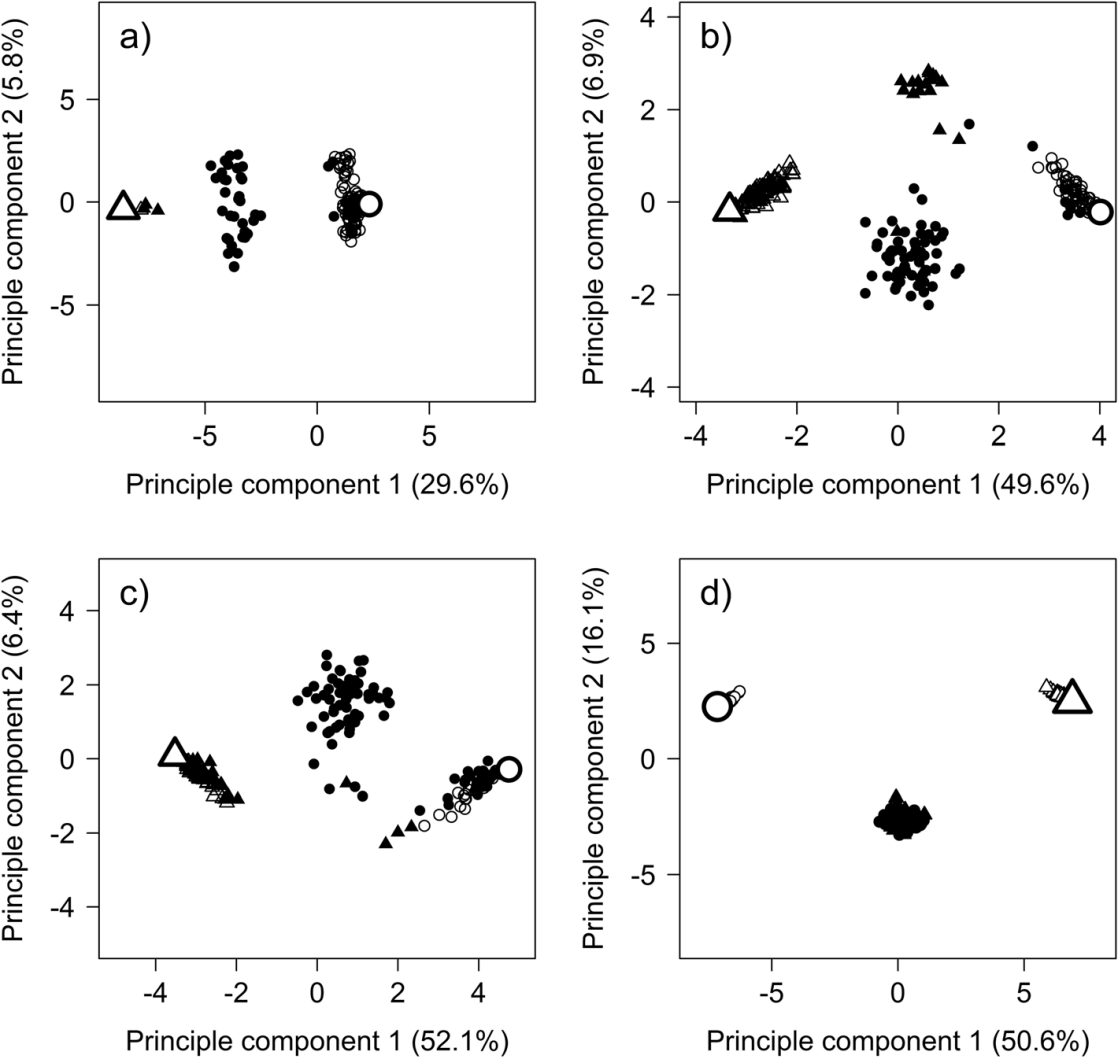
Principal component analysis of offspring from ADP populations and simulated data by SRAP marker data. Letters in brackets denote the populations: **a**) ADP 1, **b**) ADP 2, **c**) ADP 3 and **d**) simulated data. Large circles and triangles represent the two parents, small grey circles/triangles the offspring from self-fertilisation of the respective parents and small black circles/triangles the offspring from cross-fertilisation between the two parents

For the three ADP populations (Table 1), the number of markers obtained from SRAP analysis ranged from 80 to 195 (Table 3). Using these markers, high self-fertilisation rates could be identified for all three ADP populations (51.0 to 66.2 %), which were largely verified by SSR analysis (Table 3). Combined analysis using both marker systems revealed slightly lower self-fertilisation rates (48.5 to 64.8 %), because some offspring identified as selfings by SRAP markers were clearly identified as crossings based on SSR markers. Self-fertilisation rates varied within populations dependent on the maternal parent (Table 3). Principal component analysis (PCA) based on SRAP data revealed distinct grouping of offspring depending on breeding type (Fig. 1a–c). The first principal component (29.6 to 52.1 % explained variance) mainly differentiated between crossings (black symbols) and selfings (grey symbols) with the latter clustering mostly around the respective parent (Fig. 1). The second principal component (5.8 to 6.9 % explained variance) mainly separated crossings based on their parent. For all ADP populations, some of the identified crossings clustered close to the respective selfings. This may be due to the one sided nulliplex-marker evaluation for each maternal plant. We checked for non-maternal alleles at positions where the maternal plant carries the nulliplex allele, which characterise the offspring individual as crossing. Offspring with only few non-maternal alleles would be also considered as crossings, if these individuals additionally show high similarity in the non-nulliplex alleles they group closely to selfings of the respective maternal parent. However, the grouping observed was largely congruent with the one based on simulated data with 200 individuals and 200 marker loci (Fig. 1d).

**Table 3:** Self- and cross-fertilisations in populations from artificially directed pollination (ADP) determined by SRAP and SSR markers

ADP populations	Maternal subpopulations^a^	No. SRAP marker	Number of plants/selfings (selfings %)	
			SRAP	SRAP/SSR
ADP 1		195	145/96 (66.2 %)	145/94 (64.8 %)
	Visnovsky_1	104	141/95 (67.4 %)	141/93 (66.0 %)
	Perly_1	91	4/1 (25.0 %)	4/1 (25.0 %)
ADP 2		188	218/134 (61.5 %)	218/134 (61.5 %)
	Visnovsky_2	81	110/49 (44.5 %)	110/49 (44.5 %)
	Perly_2	107	108/85 (78.7 %)	108/85 (78.7 %)
ADP 3		166	237/121 (51.0 %)	237/115 (48.5 %)
	Brunner_1	86	126/34 (27.0 %)	126/30 (23.8 %)
	Perdix_1	80	111/87 (78.4 %)	111/85 (76.6 %)

^a^Maternal subpopulations originated from five clones of one single maternal plant

### Self-fertilisation in NDP populations

For the three NDP populations (Table 1), the number of SRAP markers ranged from 40 to 122 (Table 4). In these populations, generally low rates of self-fertilisation (i.e. 5.8, 0.9 and 4.5 %) were observed. After excluding potential false classifications using SSR markers, the rate of self-fertilisation decreased to 3.9 % for NDP 1, to 0.0 % for NDP 2 and to 1.8 % for NDP 3 (Table 4). NDP 1 showed the highest self-fertilisation rate characterised by SRAP markers only and combined with SSR. The self-fertilisation rate was not significantly different among NDP populations. Furthermore, no significant effect of the sampling site (corner vs. centre of the field) could be detected within NDP 2 or NDP 3.

**Table 4:** Self- and cross-fertilisations in populations from non-directed pollination (NDP) determined by SRAP and SSR markers

NDP populations	Maternal subpopulations^a^	No. SRAP markers	Number of plants/selfings (selfings %)	
			SRAP	SRAP/SSR
NDP 1		635	103/6 (5.8 %)	103/4 (3.9 %)
	NDP 1_1	86	12/0 (0.0 %)	12/0 (0.0 %)
	NDP 1_2	83	11/0 (0.0 %)	11/0 (0.0 %)
	NDP 1_3	93	13/0 (0.0 %)	13/0 (0.0 %)
	NDP 1_4	84	12/1 (8.3 %)	12/1 (8.3 %)
	NDP 1_5	62	13/1 (7.7 %)	13/0 (0.0 %)
	NDP 1_6	76	13/3 (23.1 %)	13/3 (23.1 %)
	NDP 1_7	106	15/1 (6.7 %)	15/0 (0.0 %)
	NDP 1_8	45	14/0 (0.0 %)	14/0 (0.0 %)
NDP 2		688	109/1 (0.9 %)	109/0 (0.0 %)
	NDP 2_C1	100	15/0 (0.0 %)	15/0 (0.0 %)
	NDP 2_C2	65	13/0 (0.0 %)	13/0 (0.0 %)
	NDP 2_C3	89	13/0 (0.0 %)	13/0 (0.0 %)
	NDP 2_C4	79	13/0 (0.0 %)	13/0 (0.0 %)
	NDP 2_M1	122	14/0 (0.0 %)	14/0 (0.0 %)
	NDP 2_M2	77	13/0 (0.0 %)	13/0 (0.0 %)
	NDP 2_M3	116	15/1 (6.7 %)	15/0 (0.0 %)
	NDP 2_M4	40	13/0 (0.0 %)	13/0 (0.0 %)
NDP 3		676	110/5 (4.5 %)	110/2 (1.8 %)
	NDP 3_C1	116	13/0 (0.0 %)	13/0 (0.0 %)
	NDP 3_C2	51	10/1 (10.0 %)	10/0 (0.0 %)
	NDP 3_C3	64	14/1 (7.1 %)	14/1 (7.1 %)
	NDP 3_C4	100	13/1 (7.7 %)	13/0 (0.0 %)
	NDP 3_M1	78	15/1 (6.7 %)	15/0 (0.0 %)
	NDP 3_M2	116	15/0 (0.0 %)	15/0 (0.0 %)
	NDP 3_M3	79	15/0 (0.0 %)	15/0 (0.0 %)
	NDP 3_M4	72	15/1 (6.7 %)	15/1 (6.7 %)

NDP _C sampled at the corners and _M sampled in the middle of the field site

^a^Maternal subpopulations originated from one single maternal plant

### Phenotypic characterisation of ADP populations

**Fig. 2:**
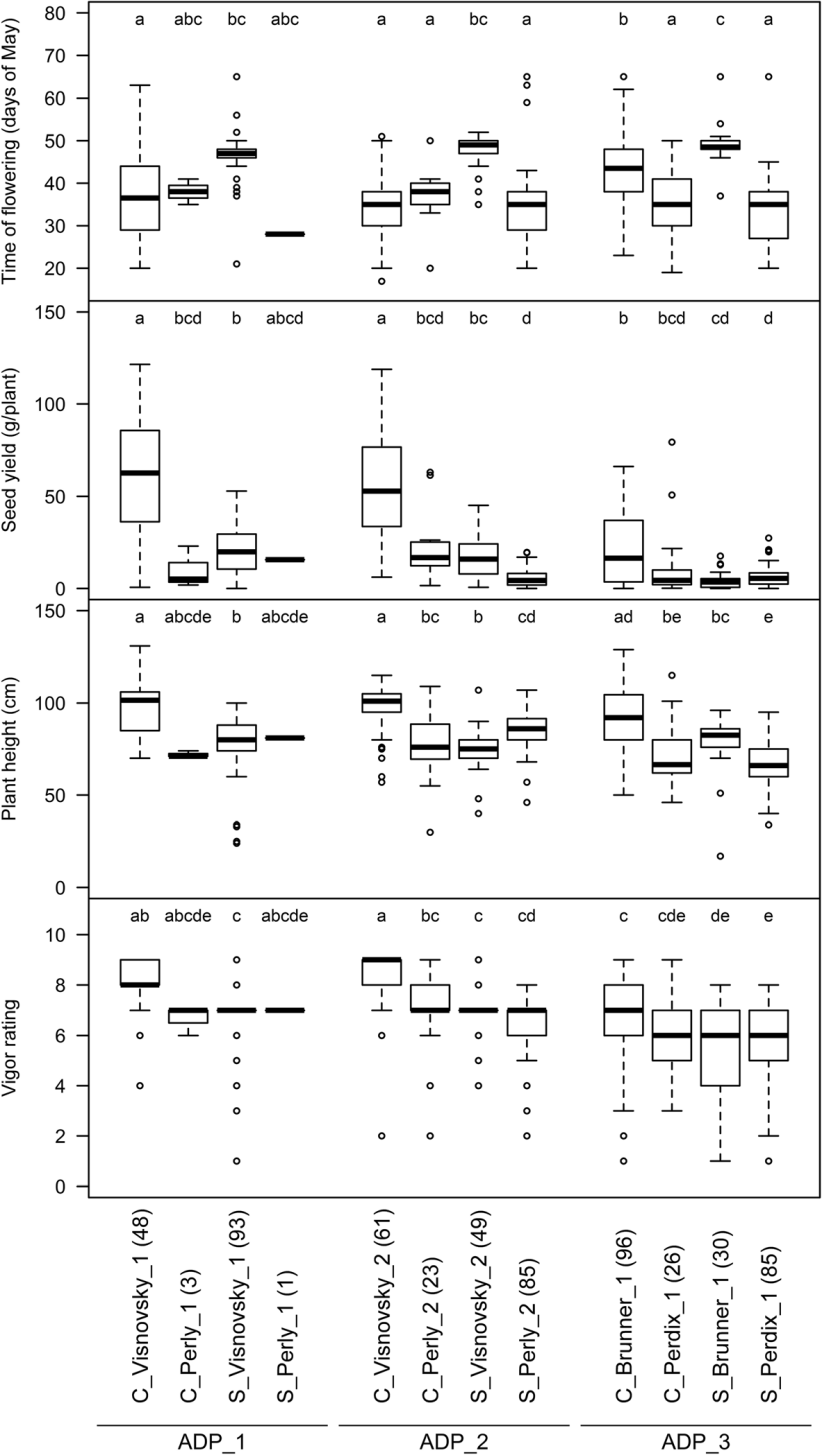
Differences of traits in populations from artificially directed pollination (ADP) dependent on cross- and self-fertilisation. C_ = offspring from cross-fertilisation; S_ = offspring from self-fertilisation. Numbers in brackets refer to the total number of plants in this group. Different letters state significant differences

The number of days until flowering for individual plants ranged from 17 days to 65 days (Fig. 2). On average, selfings of Perly_1 (28 days), Perly_2 (34 days) and Perdix_1 (33 days) showed earlier flowering than selfings from Visnovsky_1 (47 days), Visnovsky_2 (48 days) and Brunner_1 (50 days). The average flowering time for crossings ranged from 35 days to 44 days and showed less variation when compared to selfings. Overall, significant differences for flowering time were only observed between crossings from Brunner_1 and Perdix_1 and for selfings from Visnovsky_2 and Perly_2 as well as from Brunner_1 and Perdix_1. Significant differences between crossings and corresponding selfings were found for Visnovsky_1, Visnovsky_2 and Brunner_1 (Fig. 2).

Seed yield ranged from 0.0 g (plants without seed set) to 121.5 g and was mostly lower for selfings when compared to crossings. For ADP 1, mean seed yield in crossings of Visnovsky_1 was significantly reduced by 67 % in corresponding selfings. In ADP 2, selfings showed seed yields reduced by 69.4 and 70.3 % compared to crossings for Visnovsky_2 and Perly_2, respectively, the latter difference not being significant. In ADP 3, selfings showed seed yields reduced by 79.1 and 37.6 % compared to crossings for Brunner_1 and Perdix_1, the latter difference not being significant.

Overall plant height ranged from 17 to 131 cm. Average plant height was significantly lower for selfings of Visnovsky_1, Visnovsky_2 and Brunner_1 when compared to corresponding crossings (Fig. 2). In ADP 1, plant height of selfings was significantly reduced by 20.4 % for Visnovsky_1. In ADP 2, plant height of selfings was significantly reduced by 23.8 % for Visnovsky_2, but (non-significantly) increased by 10.1 % for Perly_2. In ADP 3, reductions in plant height of selfings compared to crossings ranged from 7 to 12.8 % for Perdix_1 and Brunner_1, respectively. Vigour scores, reflecting the overall performance of plants, were also affected by self-fertilisation. The scores were significantly higher in crossings with 8.1 compared to 6.7 for selfings of Visnovsky_1 and with 8.3 compared to 7 of Visnovsky_2. Across all ADP populations, breeding type, population and maternal parent, as well as their interactions had a significant influence on flowering time, seed yield, plant vigour and plant height (Table 5).

**Table 5:** Analysis of variance (ANOVA) for traits in populations from artificially directed pollination (ADP)

Phenotypic trait	Model^a^	Df	MS	F value	Pr (>F)
Flowering time	Population	2	1096.6	19.2	8.3e–9***
	Population: breeding type	3	1639.2	28.7	<2.2e–16***
	Population: parent	3	3158.2	53.4	< 2.2e–16***
	Population: breeding type: parent	3	1079.9	18.9	1.0e–11***
	Residual	567	57.0		
Seed yield	Population	2	20072.0	67.7	< 2.2e–16***
	Population: breeding type	3	40508.0	136.7	< 2.2e–16***
	Population: parent	3	8457.0	28.5	< 2.2e–16***
	Population: breeding type: parent	3	3273.0	11.0	4.7e–7***
	Residual	561	296.0		
Plant height	Population	2	3814.8	20.7	2.1e–9***
	Population: breeding type	3	10760.5	58.5	< 2.2e–16***
	Population: parent	3	4119.3	22.4	1.0e–13***
	Population: breeding type: parent	3	3940.9	21.4	3.7e–13***
	Residual	569	184.0		
Plant vigour	Population	2	60.6	31.0	1.6e–14***
	Population: breeding type	3	61.7	31.6	< 2.2e–16***
	Population: parent	3	11.7	5.9	5.2e–16***
	Population: breeding type: parent	3	6.1	3.1	0.03*
	Residual	567	1.9		

Breeding type = crossing or selfing

MS = Mean squares. * = *P* < 0.05; *** = *P* < 0.001

^a^Complete model = Population + Population: Breeding Type + Population: Breeding Type: Parent

## Discussion

### High rates of self-fertilisation can be induced

To the best of our knowledge, this is the first report on self-fertilisation rates in sainfoin under different pollination regimes and based on molecular genetic marker data. The high rates of self-fertilisation detected under artificially directed pollination (ADP) of up to 64.8 % allow for the conclusion that a strict self-incompatibility system is not functional in this species. Previous reports [15, 16] on self-fertilisation in sainfoin reported clearly lower self-fertilisation rates than those observed with ADP in this study, which might be mainly attributed to the different experimental conditions, such as plant isolation, manual or insect pollination and plant material. Under plant isolation, self-fertilisation rates of 0.98 % [16] and 1.1 % [15] were observed, which was clearly lower than the rates observed for ADP populations in this study, but comparable to rates found in non-directed pollination (NDP) populations. Following strict manual self-pollination, seed set rates of only 5.1 % [15] to 15.5 % [16] were observed, reflecting the low rates of successful self-fertilisations. In these studies, manual pollination may have been hindered by morphological barriers to self-pollination in sainfoin flowers. Open-pollination by insects in a tent with two clones of two genotypes resulted in self-fertilisation rates from 72 to 92 %, as detected using flower colour as marker [17]. This is more comparable to our findings in ADP populations. In our study, rates of self-fertilisation showed strong dependency on the maternal genotype. This could be due to a potential difference in flowering time between the two genotypes, which may have favoured self-fertilisation in earlier flowering genotypes. This is in congruence with the observation that genotypes with higher selfing rates such as Perly_2 or Perdix_1 showed earlier flowering in the field. In contrast to the ADP populations, we found very low rates of self-fertilisation in NDP populations (0 to 3.9 %), which might be caused by the large number of mature flowers on the three field sites and the ample availability of pollen from neighbouring plants. In addition, the presence of different pollinator species and their diverse activity patterns lead to a more constant pollen supply over the day, potentially decreasing the rate of self-fertilisation [10]. In order to assess whether the number of neighbouring plants influences the self-fertilisation rate, we tested for differences among sampling positions located at corners or in the middle of the fields for NDP 2 and 3, but no significant effect of the field position was found. However, self-fertilisation rates were higher in NDP 1, which was a mixed meadow with a sainfoin proportion of approximately 20 % when compared to NDP 2 and 3, which were pure stands.

### Power to detect selfings

Up to now, few sequence specific markers have been developed for sainfoin and the transferability from other species is limited [34]. Therefore, we used sequence-related amplified polymorphism (SRAP) analysis allowing to generate a large number of anonymous, dominant markers [28]. Dominant markers have been successfully used to detect self-fertilisation if marker alleles were unique in each parent [35, 36]. Unique parental alleles can be tracked in the offspring and used for the detection of cross- or self-fertilisation. The disadvantage of dominant markers is the loss of information about the genotype of an individual shown by the higher variance of estimates obtained from dominant loci compared with co-dominant loci [37]. Consequently, the dominant nature of SRAP markers makes a characterisation of self-fertilisation or cross-fertilisation ambiguous, since nulliplex genotypes can also arise from a cross of two tetraploids that are not both nulliplex, e.g. 0 0 0 0 and 1 0 0 0 (0 = allele absent, 1 = allele present) leading to false positives in the classification of self-fertilisation. However the probability of a nulliplex state after crossing two markers (0 0 0 0 × 1 0 0 0) with a probability of 0.5 for each marker locus decreases rapidly with increasing marker numbers and converges to zero with marker numbers larger than 50. In comparable studies with Caribbean corals (*Favia fragum* and *Porites astreoides*) it has been shown that 30 dominant marker were sufficient to detect all crossings [35]. Those marker numbers were lower than the marker numbers used in our study. Supplementary analysis with co-dominant SSR markers largely supported the accuracy of SRAP marker results (Table 3). A general limitation of marker fragment analysis could arise from miss-scoring fragments. However, repeated independent scoring and a large number of markers help to minimise this problem [37]. Therefore, SRAP markers demonstrated highly efficient for distinguishing offspring resulting from self- or cross fertilisations.

### Inbreeding depression dependent on trait

Plants which mainly rely on cross-fertilisation often suffer from strong decline in performance after self-fertilisation. This inbreeding depression is particularly pronounced in grassland species such as ryegrass (*Lolium perenne* L; [38, 39]) or red clover [40] with a strong self-incompatibility system. Existence and extent of inbreeding depression for sainfoin could crucially influence breeding decisions since care would have to be taken to select for genetically diverse crossing partners. Alternatively, a low inbreeding depression would allow for the development of inbred lines as a basis for hybrid breeding. Nevertheless, until now, no detailed data on inbreeding depression on plant performance was available for sainfoin. In our study, plant height and plant vigour were affected by self-fertilisation in all three ADP populations (Fig. 2). One generation of inbreeding had lowered height and vigour of selfings when compared to crossings. On the other hand, the better performance of crossings may also have been due to heterosis [41]. In our study, the decrease in performance was surprisingly strong for a potentially heterozygous tetraploid plant. In autotetraploids, recessive homozygous genotypes will be less frequent than in diploids, and inbreeding depression is expected to be lower [42]. For example, with two alleles at a frequency of 0.5 each, homozygous recessive genotypes will be present at a rate of 0.25 in diploids, but only of 0.0625 in tetraploids. Severe inbreeding depression in autotetraploids was explained by a loss of complementary gene interactions in the first few generations of inbreeding [43]. Sainfoin is a natural tetraploid, for which tri- and tetraallelic interactions are of higher importance than for artificially induced tetraploids, where diallelic interactions are predominant [44]. Such higher order interactions will be quickly lost through inbreeding, partly explaining the observed inbreeding depression for traits in the ADP populations. In addition, the high copy number in polyploids and the large genome size allow mild deleterious mutations to accumulate which can also lead to increased inbreeding depression [45]. In our study, we found that not only the breeding type significantly determined the plant height, seed yield, flowering time and vigour in all populations, but also the maternal plant influenced the plant performance (Fig. 2, Table 5), what might be attributed to different levels of heterozygosity in the maternal genotypes.

The difference in flowering time observed among plants is unlikely to influence the total seed yield, because earlier flowering does not extend the generative phase [46]. For ADP populations, a reduction in seed yield of up to 79.1 % (Fig. 2) was observed for selfings. This is remarkably high when compared to species such as alfalfa, where seed yield reductions of 55 % after one generation of inbreeding were observed [47]. Two factors could play a major role for inbreeding depression of seed yield in sainfoin. On the one hand, the fitness of the maternal plant plays an important role as seeds acts as sinks for nutrients and assimilates [48] and a good overall fitness of the maternal plant is indispensable for high seed yield. On the other hand, the possibly changed genetic composition after selfing, e.g. loss of genes or interactions and the accumulation of deleterious alleles, might have contributed to inbreeding depression. Environmental conditions may also play an important role for total seed production [49]. Our experimental setup did not allow for assessment of genotype x environment interactions, but selfings and crossings were randomly distributed across the experimental field. For flowering time, no significant difference between crossings and selfings, but a significant influence of the maternal genotype was observed (Fig. 2). Selfings from the maternal genotype Visnonsky showed the tendency of later flowering than the corresponding crossings and selfings of Perly. Selfings of Perdix showed also the same trend to earlier flowering which could be attributed to the fact that the variety Perdix originated from the variety Perly (“personal communication”, B. Boller, Agroscope Reckenholz ISS, Switzerland). Crossings showed an intermediate time of flowering reflecting the combination of genes from early and late flowering parents. This pattern of flowering time indicates additive inheritance of this trait and is in accordance with earlier studies in maize or chickpea [50, 51].

## Conclusions

This study clearly showed that a high degree of self-fertilisation could be achieved in sainfoin under controlled conditions and using insect pollination. The selfings showed significant inbreeding depression for plant height, plant vigour and seed yield. Although the dominant reproduction mechanism seems to be outbreeding, a higher rate of inbreeding can be observed under selective conditions, as they are also often present in pair- or polycross breeding schemes, i.e., open pollination within a limited set of selected elite parents. Hence, creating polycrosses composed of a sufficiently large number of parents that are strictly homogenous in flowering time is of highest importance to avoid inbreeding of the earliest genotypes. For maintenance breeding of varieties, large numbers of genotypes may help to reduce the risk of inbreeding. For targeted pair-crosses, it might become necessary to emasculate the plants which were selected as maternal parents to avoid self-fertilisation or at least to carefully check the progeny for potential selfings using genetic markers.

On the other hand, if self-fertilisation is easily accomplished, superior sainfoin varieties may be developed through hybrid breeding. For this, homogenous inbred lines from well performing and good combining genotypes have to be established and will be crossed to create a superior hybrid offspring. Therefore, our results provide a valuable basis to define strategies for the implementation of hybrid breeding in sainfoin.

The assessment of self-fertilisation in sainfoin fills a gap in knowledge of this species and the results could be applied for developing novel breeding schemes. Finally, improving underestimated species like sainfoin and integrating those plants in practical cultivation may help to enhance biodiversity in future agriculture.

## Additional files

Additional file 1: Table S1. SRAP primer combinations used for analysis of self-or cross-fertilisations in populations of artificially directed pollination (ADP) and in non-directed pollination (NDP). (PDF 113 kb) Additional file 2: Sheet S1. R-code used for simulating data of a hypothetical population consisting of crossings and selfings (Fig. 1). (PDF 327 kb)
