# Supplementary material for: Evidence and consequences of self-fertilisation in the predominantly outbreeding forage legume Onobrychis viciifolia
Source: BMC Genet. 2015 Oct 7;16:117. doi: 10.1186/s12863-015-0275-z (PMC4596305; doi:10.1186/s12863-015-0275-z)
Supplement: Additional file 1: Table S1. — SRAP primer combinations used for analysis of self-or cross-fertilisations in populations of artificially directed pollination (ADP) and in non-directed pollination (NDP). (PDF 113 kb) [file 12863_2015_275_MOESM1_ESM.pdf]

Additional file 1: Table S1

Table S1 SRAP primer combinations in populations of artificially directed pollination (ADP) and in non-directed pollination (NDP)

SRAP combinations	Forward primer (5`-3`)	Reverse primer (3`- 5`)	ADP populations	NDP populations	Reference
me1em1	TGAGTCCAAACCGGATA	GACTGCGTACGAATTAAT	✓	✓	
me2em2	TGAGTCCAAACCGGAGC	GACTGCGTACGAATTTGC	✓	✓	
me3em3	TGAGTCCAAACCGGAAT	GACTGCGTACGAATTGAC	✓	✓	
me4em4	TGAGTCCAAACCGGACC	GACTGCGTACGAATTTGA	✓	✓	
me1em2	TGAGTCCAAACCGGATA	GACTGCGTACGAATTTGC	✓	✓	
me2em3	TGAGTCCAAACCGGAGC	GACTGCGTACGAATTGAC	✓	✓	
me3em4	TGAGTCCAAACCGGAAT	GACTGCGTACGAATTTGA	✓	✓	
me4em1	TGAGTCCAAACCGGACC	GACTGCGTACGAATTAAT	✓	✓	Li and Quiros, 2001
me1em4	TGAGTCCAAACCGGATA	GACTGCGTACGAATTTGA	✓	-	
me2em1	TGAGTCCAAACCGGAGC	GACTGCGTACGAATTAAT	✓	-	
me3em2	TGAGTCCAAACCGGAAT	GACTGCGTACGAATTTGC	✓	-	
me4em3	TGAGTCCAAACCGGACC	GACTGCGTACGAATTGAC	✓	-	
me1em3	TGAGTCCAAACCGGATA	GACTGCGTACGAATTGAC	✓	-	
me2em4	TGAGTCCAAACCGGAGC	GACTGCGTACGAATTTGA	✓	-	
me3em1	TGAGTCCAAACCGGAAT	GACTGCGTACGAATTAAT	✓	-	
me4em2	TGAGTCCAAACCGGACC	GACTGCGTACGAATTTGC	✓	-	
