# Supplementary material for: Evidence and consequences of self-fertilisation in the predominantly outbreeding forage legume Onobrychis viciifolia
Source: BMC Genet. 2015 Oct 7;16:117. doi: 10.1186/s12863-015-0275-z (PMC4596305; doi:10.1186/s12863-015-0275-z)

Additional file: Sheet S1

R-code for simulation

Roland Kölliker, roland.koelliker@agroscope.admin.ch

June 4, 2015

```
##Simulation of dominant marker data for offspring of two tetraploid
hetereozygous
##plants under cross- and self-fertilization
##Written by Roland Kölliker (roland.koelliker@agroscope.admin.ch), 4.6.15
##Arguments:
##nl=number of loci
##t.bal=proportion of zeros in parental genotype
##ncross=number of crossings
##nself1=number of selfings for parent1
##nself2=number of selfings for parent2
##nco=number of recombinations,
##maxco=max number of markers per co
ffselfsim <- function(nl=200, t.bal=0.5, ncross=100, nself1=50, nself2=50,
nco=1, maxco=nl/28, ...){
  #Parental marker-phenotypes
  p1 <- sample(c(0,1), nl, replace=T, prob=c(t.bal, 1-t.bal))
  p2 <- 1-p1

  #parental genotypes
  pi1 <- NULL
  pi2 <- NULL

  #table with possible allele combinations (1,1,1,1 is not an option since
this does
  #not segregate in the progeny)
  all <- as.matrix(expand.grid (rep(list(c(0,1)), 4)))

  #create index vector
  for (i in 1:nl){
    pi1[i] <- ifelse(p1[i]==0,1, sample(2:16,1))
    pi2[i] <- ifelse(p2[i]==0,1, sample(2:16,1))
  }
  #create parental genotypes
  pa1 <- all[pi1,]
  pa2 <- all[pi2,]

  cbind(p1,pa1)
  cbind(p2,pa2)
```

```
  #function to create gametes with recombination

  #(t.x <- matrix(rep(c("A","B","C","D"), each=nl), ncol=4)) #test to see
whether this works

  ffgam <- function(x) {
    t.g1 <- x[, sample(1:4,4)]
    tt.g1 <- t.g1
    for (i in 1:nco){
      anco1 <- sample(maxco,1) #number of markers concerned
      loco1 <- sample(1:(nl-anco1),1) #locatin of co
      anco2 <- sample(maxco,1) #number of markers concerned
      loco2 <- sample(1:(nl-anco2),1) #locatin of co

      tt.g1[loco1:(loco1+anco1),1] <- t.g1[loco1:(loco1+anco1),2]
      tt.g1[loco1:(loco1+anco1),2] <- t.g1[loco1:(loco1+anco1),1]
      tt.g1[loco2:(loco2+anco2),3] <- t.g1[loco2:(loco2+anco2),4]
      tt.g1[loco2:(loco2+anco2),4] <- t.g1[loco2:(loco2+anco2),3]
      ga1 <- cbind(tt.g1[,sample(1:2,1)], tt.g1[,sample(3:4,1)])
    }
    return(ga1)
  }

  ##create genotypes of crossings for autopolyploids
  ntot <- ncross+nself1+nself2

  d.prog <- matrix(nrow=ntot, ncol=nl)
  for (i in 1:ncross){
    c.auto <- cbind(ffgam(pa1), ffgam(pa2))
    d.prog[i,] <- apply(c.auto,1, function(x) ifelse(any(x==1),1,0))
  }
  #selfings parent 1
  for (i in (ncross+1):(ncross+nself1)){
    c.auto <- cbind(ffgam(pa1), ffgam(pa1))
    d.prog[i,] <- apply(c.auto,1, function(x) ifelse(any(x==1),1,0))
  }
  #selfings parent2
  for (i in (ncross+nself1+1):ntot){
    c.auto <- cbind(ffgam(pa2), ffgam(pa2))
    d.prog[i,] <- apply(c.auto,1, function(x) ifelse(any(x==1),1,0))
  }
  d.prog <- data.frame(d.prog)
  rownames(d.prog) <- c(paste0("C",1:ncross),
paste0("SA",(ncross+1):(ncross+nself1)), paste0("SB",(ncross+nself1+1):ntot))
  head(d.prog)
  d.final <- rbind(p1, p2,d.prog)
  head(d.final)
```

```
  r.pca <- prcomp(d.final)
  plot(r.pca$x, pch=c(21,22,rep(23,ncross),rep(21,nself1), rep(22,nself2)),
bg=c(2,3,rep(1,ncross), rep(2,nself1), rep(3,nself2)),
cex=c(3,3,rep(1.3,ntot)), ...)
}

set.seed(1895)
ffselfsim(main="Simulated data for 200 markers,\n 200 individuals and 50
selfings each", xlim=c(-8,8), ylim=c(-8,8))
```

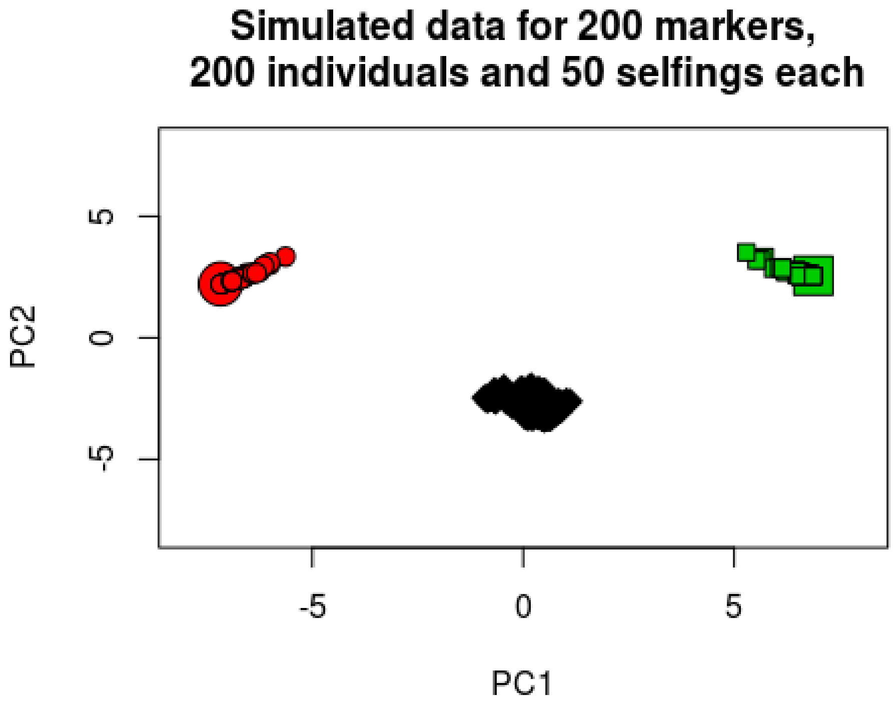

Supplement: Additional file 2: Sheet S1. — R-code used for simulating data of a hypothetical population consisting of crossings and selfings (Fig. 1). (PDF 327 kb) [file 12863_2015_275_MOESM2_ESM.pdf]
